# Mast Cells in Immune-Mediated Cholangitis and Cholangiocarcinoma

**DOI:** 10.3390/cells11030375

**Published:** 2022-01-22

**Authors:** Marisol I. González, Danielle T. Vannan, Bertus Eksteen, Irán Flores-Sotelo, José Luis Reyes

**Affiliations:** 1 Laboratorio de Inmunología Experimental y Regulación de la Inflamación Hepato-intestinal, UBIMED, FES Iztacala, UNAM, Tlalnepantla de Baz 54090, State of Mexico, Mexico; migonzalez@iztacala.unam.mx (M.I.G.); warceli_2000@hotmail.com (I.F.-S.); 2 Endoscopy Division, Boston Scientific Corporation, 200 Boston Scientific Way, Marlborough, MA 01752-1234, USA; daniellevannan@gmail.com; 3 Aspen Woods Clinic, Calgary, AB T3H 0V5, Canada; bertus@aspenwoodsclinic.com

**Keywords:** mast cells, cholangitis, cholangiocarcinoma, PBC, PSC, liver fibrosis

## Abstract

Cholestasis, which is impaired bile flow from the liver into the intestine, can be caused by cholangitis and/or bile duct obstruction. Cholangitis can arise from bacterial infections and cholelithiasis, however, immune-mediated cholangitis in primary biliary cholangitis (PBC) and primary sclerosing cholangitis (PSC) is characterized by a strong immune response targeting the biliary epithelial cells (BECs). Persistent biliary inflammation further represents a risk for biliary neoplasia, cholangiocarcinoma (CCA) by driving chronic cellular stress in the BECs. Currently, immune-mediated cholangitis is considered a Th1-Th17-dominant disease, however, the presence of Th2-related mast cells (MCs) in tissue samples from PBC, PSC and CCA patients has been described, showing that these MCs are active players in these diseases. Here, we reviewed and discussed experimental and clinical data supporting a pro-fibrotic role for MCs in immune-mediated cholangitis as well as their participation in supporting tumor growth acting as angiogenesis promoters. Thus, although MCs have classically been identified as downstream effectors of Th2 responses in allergies and parasitic infections, evidence suggests that these MCs are relevant players in biliary inflammation and neoplasia. The availability of strategies to prevent MCs’ activation represents a therapeutic opportunity in biliary diseases.

## 1. Immunobiology of Mast Cells

Mast cells (MCs) are a cell lineage produced in the bone marrow from myeloid precursors which express and retain c-kit expression throughout their developmental stages [1]. In order to expand MC population signals delivered from stem cell factor (SCF), the c-kit ligand is required; although, it has been shown that additional players such as interleukin (IL)-3 and nerve growth factor (NGF) can also promote in vitro MC growth [2,3]. MCs incompletely differentiated egress from bone marrow as evidenced by the absence of granules in circulating committed MCs. However, once they enter the tissues of the body, cell maturation takes place [1,4]. In terms of location, MCs lay beneath vascular endothelial cells (blood vessels), nerves and epithelium-covered surfaces (skin, airways, urinary and gastrointestinal tracts) which enables them to act as first responders in settings such as trauma and thermal injury. Furthermore, as in many other immune cell populations, MCs are a heterogeneous cell type which is subdivided based on location and granule content. Mast cells are typically classified into two types: connective tissue type and mucosal type. In mice, they are called connective tissue mast cells (CTMCs) and mucosal mast cells (MMCs), respectively. In humans, mast cells that contain both tryptase and chymase (MC_TC_) correspond to CTMCs in mice, and mast cells that contain tryptase and no chymase (MC_T_) correspond to MMCs in mice [1]. Moreover, it has been reported that MCs are able to regulate their enzyme expression in a highly dynamic manner depending on the phase of infection in the same tissue [5] and specific stimuli, such as helminthic parasites which trigger massive MC expansion [6]. Thus, salient evidence suggests a complex compartmentalization of heterogeneous populations of MCs in the immune system of mammals. 

In terms of MC response, these cells are activated by immune complexes (antigens bound to IgG and mostly IgE), complement products (C3a and C5a), ligation of pattern recognition receptors (PRRs) on their surface and interestingly, these cells are activated by venoms from poisonous animals. Once activated, MCs release a great array of mediators such as Th2-promoting (IL-4) and inflammatory cytokines such as interleukin (IL)-1β and tumor necrosis factor (TNF)-α, lipidic inflammatory mediators (leukotrienes) and more importantly, MCs promote pathological conditions by releasing pro-fibrotic mediators such as transforming growth factor (TGF)-β and fibroblast growth factor (FGF) [7,8]. Additionally, tryptase, one of the main proteases stored in MC granules can activate and induce the proliferation of fibroblasts [9,10]. It is worth noting that MCs are known as the primary source of histamine which plays a critical role in allergies as pro-angiogenic factor. This variety of responses enables MCs to initiate and modulate activation of the immune system under different contexts (allergy, infections, inflammation and cancer) and place them as critical early and late players of the immune response.

## 2. Mast Cells in the Hepatopancreaticobiliary (HPB) System

The liver and pancreas are interconnected with the gallbladder and the biliary tree through an integrated network of bile ducts known as the hepatopancreaticobiliary (HPB) system. Interestingly, MCs are located throughout the HPB system and, under physiological conditions, contribute to the maintenance of homeostasis. Pioneer studies reported MCs residing in low numbers in the human liver [11] and it is now known that MCs are actually found in periportal spaces [12], beneath the biliary epithelial cells (BECs) (peribiliary) [13] and surrounding the sinusoids [14]. In these sites, tryptase-positive MCs were found to be in close contact with vascular smooth muscle cells and proposed to be involved in regulating the vascular plexus surrounding the bile ducts and blood flow in normal and cirrhotic livers, likely via nitric oxide (NO), endothelin1 (ET-1) and chymase [13]. Interestingly, there seems to be a complementary interplay amongst MCs, BECs and peripheral nerves since BECs influence MCs’ activation through stem cell factor expression [15] and nerve cells can also modulate histamine release from MCs [16]. Solid evidence shows that MCs are active players in autoimmune liver diseases (e.g., hepatitis) [17] and can also support tumor growth in hepatocellular carcinoma by expanding suppressive cells which in fact results in a poor prognosis [18,19,20]. Moreover, the density of MCs in liver samples increases in biliary diseases, as we will discuss in the following sections.

It has been reported that MCs also inhabit the gallbladder in both pediatric [21] and adult populations [22]. MC-specific detection was achieved by means of tryptase and CD117 (c-kit) staining. Interestingly, in the pediatric study, authors showed a maximum average of 2.25 MCs per high-power field (HPF), whereas adult samples presented at least 20 MCs per HPF on average. As expected, the density of MCs was dramatically increased in biliary diseases such as biliary dyskinesia and cholelithiasis in pediatric patients [21] whereas in adult populations the increase in MC density was modest but significant as well [22]. Recently, additional evidence confirmed high density of MCs in gallbladder samples from patients with biliary dyskinesia and cholelithiasis [23]. These findings are intriguing given that it has long been identified that histamine causes gallbladder contraction [24] and these diseases are part of the functional gallbladder disorder where gallbladder motility is altered. Thus, the reason why high density of MCs correlated with altered motility remains to be determined.

The presence of MCs in pancreatic samples from healthy individuals has also been observed. Cells staining positive for tryptase and chymase were observed in interstitial spaces between acini and connective tissue and surrounding blood vessels and nerves from healthy donors, where the average numbers were reported to be 7.6 MCs [25], which is different from those numbers reported in gallbladder [21,22]. Interestingly, numbers of MCs were found to be increased in pancreatic pathologies such as chronic pancreatitis (CP) [25] and pancreatic neoplasia, including pancreatic ductular adenocarcinoma (PDAC) [26] and islet cell tumors [27]. In line with liver diseases, MCs accumulate in fibrotic areas [25] and in PDAC biopsies were found to be significantly concentrated in the intratumoral border zone. In addition, MC counts were positively associated with microvessel proliferation [26,28]. 

Therefore, MCs can be considered a resident cell type in the HPB system, and their density depends on several factors such as anatomic site and age. A great expansion of MCs in chronic stages of diverse HPB diseases is evident with a topographical selectivity.

## 3. Mast Cells in Immune-Mediated Cholangitis

Immune-mediated cholangitis in primary biliary cholangitis (PBC) and primary sclerosing cholangitis (PSC) is characterized as a sustained inflammatory response targeting the biliary epithelial cells (BECs) causing an initial disarrangement and subsequent narrowing of the bile ducts due to fibrotic remodeling. Subsequently, chronic inflammation may lead to liver failure and/or malignancy. Both PBC and PSC share similarities such as diagnostic images where obliteration of the bile ducts is easily observed showing a beading pattern and histological findings unveiling both infiltrating cells and collagen fibers deposited around the bile ducts. In addition, PBC and PSC patients present biochemical and tissue architecture modifications in the gallbladder [29,30]. However, these diseases are not fully similar. In general, PBC is associated with autoimmune diseases (lupus, rheumatic and autoimmune hepatitis) and it occurs predominantly in women [31], high rates of patients show increased levels of circulating auto-antibodies including antinuclear (ANA) and antimitochondrial (AMA) [32]. Moreover, to date several identified genetic risk factors for PBC have been found in autoimmune-related loci such as HLA class II genes as well as in non-HLA alleles (e.g., *NF-κB*, *SOCS1*, *STAT4*) [33,34]. In terms of PSC, circulating auto-antibodies are much less frequent and this disease is strongly associated with autoinflammatory diseases (i.e., ulcerative colitis) rather than autoimmune counterparts [35]. Additionally, men are more affected than women. Further, unlike PBC, PSC is the strongest clinical event associated with cholangiocarcinoma (CCA).

While both PBC and PSC have been identified as Th1- and Th17-related diseases, they also share the pathological component of fibrotic reactions resulting from chronic inflammation. Fibrotic reactions are largely promoted and sustained by components of the Th2 response, including MCs. In fact, it has been long identified that MCs release an array of pro-fibrotic mediators including IL-1β, TNFα, TGFβ and FGF, which can also be produced by other sources. Additionally, there exists several MC-specific pro-fibrotic molecules such as histamine, tryptase and chymase. It is worth noting that elevated levels of histamine are reported in chronic cholestatic diseases and are associated with the major symptom of these diseases, which is pruritus [36]. Moreover, histamine evokes collagen production by fibroblasts [37]. Therefore, evidence suggests an important role of MCs in immune cholangitis.

Both clinical and experimental evidence has emerged showing that MCs accumulate in the biliary tree and gallbladder in PBC and PSC patients in higher numbers compared to acute liver diseases and other chronic liver diseases. Clinical data have, for instance, shown that patients with PBC had increased numbers of MCs, determined by tryptase staining, as part of infiltrating cells in portal tracts [12,38,39,40]. Importantly, MCs were detected in zones of fibrosis in diverse hepatic areas such as fibrotic portal tracts and fibrotic septa [40]. Likewise, tissue samples from PSC patients were positive for tryptase and c-kit staining confirming the presence of MCs in the portal tracts, where a positive association between MC numbers and PSC severity was found [41,42,43,44]. Moreover, PSC patients had overexpression of SCF on the BECs [42], suggesting that cholangiocytes may actively be promoting activation and proliferation of MCs. Complementarily, animal studies have shown that MCs can communicate with hepatic stellate cells (HSCs) through TGFβ which induces a profibrotic program in HSCs (i.e., alpha smooth muscle actin, αSMA induction) [45]. Thus, these observations suggest that a crosstalk between BECs and MCs in chronic PBC and PSC may be inducing fibroblast and HSCs activation causing fibrotic reactions.

Despite mouse models of immune cholangitis being difficult to establish due to complexity in these diseases, relevant insight into immunopathology as well as preclinical therapeutic evaluation has been leveraged using animal models. Adding to the human studies, mouse model research has confirmed that MCs represent one important immune cell population expanding in the injured bile ducts. Using the multidrug resistant gene 2 deficient mouse model (Mdr2^−/−^), Jones et al. showed that, in this spontaneous model of PSC, a significant increase in MCs paralleled the biliary injury. Moreover, when MCs were inhibited with cromolyn sodium, a significant decrease in biliary injury, cholangiocyte proliferation and collagen deposition was observed. The latter was associated with lower MC numbers and circulating histamine levels [44]. More recently, the same research group described that, amongst other mechanisms, the FDA approved treatment for cholestatic diseases ursodeoxycholic acid (UDCA) similarly attenuates MC infiltration, fibrosis, and histamine secretion [43]. Interestingly, in these studies, using samples from PSC patients complemented the mouse results, providing evidence that controlling MC activity is part of the immune cholangitis treatment highlighting the relevance of these cells.

As mentioned earlier, PBC meets the criteria to be considered as an autoimmune disease, although a mouse model reproducing the full hallmarks for this disease is not yet available. However, the OVAbil model [46], induced in an antigen-specific and cholangiocyte-restricted fashion reproduces the biliary damage observed in both PBC and PSC and is highly dependent on a progressive immune response which ultimately damages the bile ducts. Therefore, the OVAbil model closely resembles autoimmune-mediated cholangitis rather than other chemical injury-induced cholangitis models. Using this model, we observed that obese mice and obese mice lacking the sensor NLRP3, both presented a more severe form of the disease, had a mixed Th2/Th17 (IL-13 and IL-17) response and IL-13, respectively [47,48]. Interestingly, other granulocytes such as neutrophils massively infiltrated in the liver. Whether Th2 downstream effectors such as MCs indeed collaborated in exacerbating cholangitis in these OVAbil obese mice remains to be tested.

Thus, convincing evidence shows that MC expansion accompanies the development of immune cholangitis (i.e., PBC and PSC) and these cells promote fibrotic reactions by communicating with other resident liver cells including HSCs and fibroblasts which worsen the course of PBC and PSC. Impaired MC infiltration and histamine release results in attenuated biliary injury. Summarized evidence showing the role of MCs in immune cholangitis is presented in Table 1 where studies retrieved from a literature search in the PUBMED database including the keywords mouse, human, cholangitis, and mast cells were included with no time period restriction.

## 4. Immune Cholangitis Treatment Targets Mast Cells

Up to now, the most effective FDA-approved treatment for biliary diseases such as chronic cholestatic PBC and PSC as well as in gallbladder contractility disorders is ursodeoxycholic acid (UDCA). Interest in knowing how this treatment exerts its anti-cholestatic effects has revealed that, in addition to reducing the cytotoxic effect of bile acids, UDCA indeed evokes a concomitant anti-inflammatory immune response. UDCA treatment has been shown to diminish macrophage infiltration and MC degranulation as well as oxidative stress in patients with gallbladder cholesterol gallstones [49]. The anti-inflammatory effect of UDCA also includes control of MCs. Carotti et. al. showed that UDCA delivery also reduced the number of degranulated MCs which restored gallbladder contractility [49]. In line with this, Meng et. al. addressed the effect of UDCA on Mdr2^−/−^ mice (PSC model) and PSC patients. The authors found that both Mdr2 deficient mice and patients with PSC exposed to UDCA presented reduced signs of biliary injury such as portal inflammation and necrosis as well as ductular reaction. UDCA supplementation caused lower numbers of MCs and less fibrosis, as compared to individuals without UDCA diet enrichment [43]. Interestingly, the superior effectiveness of UDCA on controlling Th2-type inflammation in liver diseases has prompted its use as a Th2 regulator in lung diseases with promising results as gauged in asthma models where UDCA attenuated lung inflammation including reduced mast cell numbers [50]. Finally, several reports have recently described that UDCA treatment indeed controlled the inflammatory cytokine storm in COVID 19 patients evidencing the role of UDCA as an immunomodulator agent [51,52].

## 5. Mast Cells in Cholangiocarcinoma

Cholangiocarcinoma (CCA) constitutes a group of cancers occurring in diverse parts of the biliary system (intrahepatic (iCCA) and extrahepatic (eCCA)) with several origins (adenocarcinomas and adenosquamous) and type-specific epidemiology (iCCA shows an increasing rate whereas eCCA seems to be more stable) [53,54]. Additionally, in some parts of the globe such as East Asia, CCA presents a high prevalence, which is related to biliary inflammation caused by chronic helminthic infections. By contrast, in western countries CCA is related to chronic non-infectious inflammatory diseases such as PSC. CCA is divided into fluke related CCA and non-fluke related CCA [55]. 

It has been shown that tumor cells, including cholangiocytes have the ability of secreting SCF (c-kit ligand) [56], therefore biliary tumor cells recruit infiltrating MCs, which are components of the biliary tract tumor environment. In fact, MCs are significantly increased in CCA human samples as compared to non-malignant or normal liver tissue samples [56,57]. However, conflicting results suggest that MCs may be displaying a dual role in carcinogenesis. For instance, c-kit^+^ cells were detected in low frequencies in iCCA and eCCa samples in German patients’ cohort and the highest significance as predictor was assessed for adaptive cells [58]. Bo et al. showed that tumor infiltrating MCs (tryptase positive cells) were associated with anti-tumor CD8^+^ T cells and, in turn, with prolonged overall survival (OS) [59]. In line with this, higher densities of MCs in gallbladder cancer samples were associated with supporting CD8^+^ T cell responses improving the effectiveness of chemotherapy [60]. Thus, MCs turned out to be a favorable prognostic factor in biliary tract cancer patients, including CCA, within a Chinese cohort [59]. 

On the other hand, several reports suggest that MCs rather promote CCA cells growth through histamine release. Mice implanted with a CCA cell line (Mz-ChA-1) receiving histamine showed increased MC infiltration. Conversely, mice given an MC stabilizer (cromolyn sodium) showed less tumor growth. Interestingly, the authors found that histamine treatment evoked angiogenesis by means, partly, of VEGF expression [56]. Additionally, CCA cells (Mz-ChA-1) exposed to MC supernatants enhanced gene transcripts of matrix metalloprotease (*MMP*) -2, -3 and -9, as well as *VIMENTIN*, *PAXILLIN* and *S100A4.* Thus, it was demonstrated that MCs have the ability of providing a permissive environment for CCA cell growth. To confirm this, the administration of histamine receptor antagonists reduced cholangiocyte proliferation and CCA tumor growth [61]. These above-mentioned discrepancies might be related to microenvironment-dependent subtypes of MCs since MCs have also been subdivided as MC1 and MC2, according to their anti-tumorigenic and pro-tumorigenic features [62]. Table 2 shows studies obtained from the PUBMED database under the keywords search for cholangiocarcinoma and mast cells with no period restriction describing the role so far attributed to MCs during CCA.

## 6. Conclusions

Mast cells have traditionally been associated with Th2 diseases such as allergies and parasitic infections. However, as discussed here, current evidence describes the presence of MCs in the HPB system where they are able to integrate signals from BECs and to secrete cytokines and growth factors in response. Furthermore, these immune mediators can act back on BECs, fibroblasts and HSC, thus creating bidirectional communicating loops within the HPB system. This complex network results in sustained pro-fibrotic reactions in chronic cholestatic diseases (PBC and PSC), in part promoted by MCs. Hence, the effectiveness of UDCA treatment partly relies on its inhibitory function over MCs. In biliary neoplasia (CCA), an incomplete landscape suggests dual roles of MCs either context-dependent or stage-dependent. The development of therapeutics that target MCs may represent a novel treatment opportunity in chronic immune-mediated cholestatic liver disease.

## Figures and Tables

**Table 1:** Relevance of MCs in human and mouse immune cholangitis.

Host/Model	MC subtype	Location	Disease outcome	Ref.
Human PBC	Tryptase ^+^	Portal tracts	N.D.	[39]
Human PBC	Tryptase ^+^	Hepatic lobules with no significant increase Significant increase in fibrotic small portal tracts	Putative fibrosis promoters	[38]
Human PBC	Chymase ^+^	Fibrotic portal areas	N.D.	[40]
Human PSC	Tryptase ^+^	Hepatic lobules with no significant increase Significant increase in fibrotic small portal tracts	Putative fibrosis promoters	[38]
Human PSC	Tryptase^+^	Bile ducts	Fibroplasia and inflammation	[42]
Human PSC	Tryptase^+^	Bile ducts	Bile duct obstruction	[43]
Mouse PSC (Mdr2^−/−^ model)	mMCP-1	Bile ducts	Fibrosis and biliary proliferation	[43]
Mouse PSC (Mdr2^−/−^ model)	Chymase^+^	Bile ducts	Hepatic fibrosis	[44]

Abbreviations: mMCP-1, mouse mast cell protease 1; Mdr2^−/−^, multidrug resistant gene 2 deficient mice; N.D., not determined; PBC, primary biliary cirrhosis; PSC, primary sclerosing cholangitis.

**Table 2:** Diverse roles of MCs in cholangiocarcinoma.

Host/Model	MC subtype	Location	Disease outcome	Ref.
Human iCCA/eCCA	CD117 (c kit)^+^	Tumor microenvironment	Not significant role as predictor	[58]
Human eCCA (pCCA and dCCA)	Tryptase^+^	Tumor stroma	Tumor MCs correlated with favorable prognosis and improved response to gemcitabine therapy	[59]
Human iCCA	Tryptase^+^ and Tryptase^+^ Chymase^+^	Tumor stroma	N.D.	[60]
Human CCA	N.D.	N.D.	Inhibition of histamine receptor reduced BECs proliferation	[61]
Human CCA	c-kit^+^, Tryptase^+^ and Chymase^+^	Tumor stroma	N.D.	[56]
Mouse CCA	Tryptase^+^ and Chymase^+^	Tumor stroma	MCs targeting with cromolyn sodium reduced tumor size and genes associated to EMT	[56]
Mouse CCA	Toluidine stain^+^	Tumor stroma	Histamine receptor blockade reduced tumor size	[61]

Abbreviations: eCCA, extrahepatic cholangiocarcinoma; iCCA, intrahepatic cholangiocarcinoma; pCCA, perihilar cholangiocarcinoma and dCCA, distal cholangiocarcinoma.
